# Old Players with a Newly Defined Function: Fra-1 and c-Fos Support Growth of Human Malignant Breast Tumors by Activating Membrane Biogenesis at the Cytoplasm

**DOI:** 10.1371/journal.pone.0053211

**Published:** 2013-01-02

**Authors:** Ruben D. Motrich, Gonzalo M. Castro, Beatriz L. Caputto

**Affiliations:** Centro de Investigaciones en Química Biológica de Córdoba, (Universidad Nacional de Córdoba-The National Scientific and Technical Research Council), Departamento de Química Biológica, Facultad de Ciencias Químicas, Universidad Nacional de Córdoba, Ciudad Universitaria, Córdoba, Argentina; University of Saarland Medical School, Germany

## Abstract

A shared characteristic of tumor cells is their exacerbated growth. Consequently, tumor cells demand high rates of phospholipid synthesis required for membrane biogenesis to support their growth. c-Fos, in addition to its AP-1 transcription factor activity, is the only protein known up to date that is capable of activating lipid synthesis in normal and brain tumor tissue. For this latter activity, c-Fos associates to the endoplasmic reticulum (ER) through its N-terminal domain and activates phospholipid synthesis, an event that requires it Basic Domain (BD) (aa 139–159). Fra-1, another member of the FOS family of proteins, is over-expressed in human breast cancer cells and its BD is highly homologous to that of c-Fos with two conservative substitutions in its basic amino acids. Consequently, herein we examined if Fra-1 and/or c-Fos participate in growth of breast cancer cells by activating phospholipid synthesis as found previously for c-Fos in brain tumors. We found both Fra-1 and c-Fos over-expressed in >95% of human ductal breast carcinoma biopsies examined contrasting with the very low or undetectable levels in normal tissue. Furthermore, both proteins associate to the ER and activate phospholipid synthesis in cultured MCF7 and MDA-MB231 breast cancer cells and in human breast cancer samples. Stripping tumor membranes of Fra-1 and c-Fos prior to assaying their lipid synthesis capacity *in vitro* results in non-activated lipid synthesis levels that are restored to their initial activated state by addition of Fra-1 and/or c-Fos to the assays. In MDA-MB231 cells primed to proliferate, blocking Fra-1 and c-Fos with neutralizing antibodies blocks lipid-synthesis activation and cells do not proliferate. Taken together, these results disclose the cytoplasmic activity of Fra-1 and c-Fos as potential targets for controlling growth of breast carcinomas by decreasing the rate of membrane biogenesis required for growth.

## Introduction

The *fos* and *jun* oncogenes are members of the family of Immediate Early Genes (IEGs) AP-1 transcription factors that are rapidly and transiently expressed in different cell types in response to a myriad of stimuli, such as growth factors, neurotransmitters, etc. [1]–[3]. The Fos proteins (c-Fos, Fra-1, Fra-2 and Fos-B) and the Jun proteins (c-Jun, JunB and JunD) share homologous regions containing a basic DNA-binding domain (BD) and a leucine zipper dimerization motif. Jun proteins form homo- and heterodimers whereas Fos proteins only form heterodimers with other IEG´s, mostly Jun proteins, thus originating the variety of AP-1 transcription factors that regulate target genes expression in response to growth factors [1], [4].

Although c-Fos was described as an AP-1 transcription factor more than 20 years ago, the complex consequences of its induction on cell physiology have still not been fully elucidated. It has been proposed that, upon mitogenic stimuli, c-Fos triggers and controls cell growth, differentiation and apoptosis by regulating key genes [5]. However, we have shown that in addition to its nuclear AP-1 activity, c-Fos associates to the endoplasmic reticulum (ER) and activates phospholipid synthesis as an additional response to mitogenic stimuli [6]. This cytoplasmic activity of c-Fos has been observed *in vivo* in light-stimulated retina ganglion and photoreceptor cells [6]–[9], in culture in NIH3T3 fibroblasts induced to re-enter growth [10], in PC12 cells induced to differentiate [11], [12], in actively growing and proliferating T98G glioblastoma multiforme-derived cells [13], [14], and in human and mouse tumors from the Peripheral and Central Nervous Systems [15], [16]. Although the mechanism by which c-Fos associates to the ER and activates phospholipid biosynthesis is currently not fully elucidated, it is known that c-Fos physically associates with specific, key enzymes of the pathway of phospholipid synthesis in the ER [17]. c-Fos/ER association is regulated by the phosphorylation state of c-Fos-tyrosine residues #10 and #30 whereas its activation capacity depends on its BD (20 amino acids spanning from 139–159) [13], [14], [17].

The expression of Fra-1 (the Fos-related antigen-1), another member of the Fos family of proteins, is encoded by the fos-like-1 gene (*fosl1*) is also induced by mitogenic stimuli [18], [19]; its role in cell growth is also not well understood. Fra-1-AP-1 transcriptional activity is regulated both transcriptionally [20]–[22] and post-translationally [22]. In exponentially growing cells, Fra-1 expression is elevated and it is hyper-phosphorylated on C-terminus Ser and Thr residues [23]. These phosphorylation sites seem to play a role both in stabilizing Fra-1 and on its AP-1 activity [22]–[26]. In spite that the leucine zipper domain of Fra-1 is homologous to that of other Fos family members, transcriptional activation studies suggest that Fra-1 is a negative regulator of AP-1 activity [25], [26].

Among the Fos family members, Fra-1 is probably the most frequently expressed in different forms of human cancer [22], [27]: its over-expression has been reported in proliferative disorders such as breast, lung, colon, oesophageal, brain and thyroid cancer [22], [27]–[38]. Fra-1 has also been shown as a feature of hyperplastic and neoplastic breast disorders [31]–[33] with capacity to regulate proliferation and invasiveness of breast cancer cells [29], [30]. A positive correlation has been found between Fra-1 expression and an aggressive phenotype in breast invasive ductal carcinoma [34]. However, most work has focused on studying the nuclear activity of Fra-1 in spite that its cytoplasmic over-expression has been reported in breast, lung and thyroid cancer [31], [33]–[35], [38].

Tumor cells demand high rates of phospholipid synthesis to support membrane biogenesis for their exacerbated growth. In mice knock-out for c-Fos and knock-in for Fra-1 under the c-Fos promoter, Fra-1 rescues and substitutes for growth-dependent functions of c-Fos but not for its AP-1 activity [39], suggesting that Fra-1 and c-Fos may substitute each other in some of their biological functions. Herein we examined Fra-1 and c-Fos expression in growing breast cancer cells and their capacity to activate phospholipid synthesis. It is shown that both Fra-1 and c-Fos are over-expressed in >95% of ductal breast carcinoma tissue samples contrasting with very low or undetectable levels in normal tissue. Both Fra-1 and c-Fos were found associated to the ER and activating phospholipid synthesis in breast cancer cells in culture and in human breast cancer tissue samples, thus disclosing this activity of these proteins as a potential target for controlling growth of breast carcinomas.

## Materials and Methods

### Ethics Statement

Freshly excised human breast tumor and matched benign specimens were obtained from female patients with written consent following the procedures indicated by the Research Ethics Board of the Hospital Nacional de Clínicas, Universidad Nacional de Córdoba, Argentina, and with the Helsinki Declaration of 1975, as revised in 1983. Samples were processed anonymously. Patient ages ranged from 38–82 years old. All the procedures used for this study were approved by the Ethics Committee of CIQUIBIC-CONICET.

### Cell Cultures and Extracts

MCF7 and MDA-MB231 cells (ATCC-Bethesda, MD, USA) were grown under standard culture conditions in Dulbecco’s modified Eagle medium (Gibco, BRL, Invitrogen, Carlsbad, CA, USA) supplemented with 10% foetal bovine serum (FBS). After desired confluence, growth was continued for 36 h (MCF7 cells) or 72 h (MDA-MB231 cells) –FBS to achieve quiescence. Cells re-entered growth by addition of 10% FBS or cultures continued –FBS, as indicated.

Cells were transfected 100 µg/ml Fra-1 or c-Fos antibody (Santa Cruz Biotechnology, Santa Cruz, CA, USA), using the BioPORTER QuickEasy Protein Delivery Kit (Sigma-Aldrich, Saint Louis, MO, USA). Transfection efficiency >75% was controlled using FITC-IgG antibody. AP-1 nuclear localization signal peptide (NLSP) (American Peptide Company, Sunnyvale, CA, USA) that blocks AP-1 nuclear import [11] was added to cultures at 75 µM final concentration in 5 µl of medium. Control cells received the same volume of delivery medium.

### Sub-cellular Fractionation

This was as previously described [13]. Briefly, total homogenates (TH) (rinsed cultured cells or tissue samples) prepared in PBS plus protease inhibitor cocktail (Sigma-Aldrich), were processed with an Ultra-turrax homogenizer and centrifuged for 1 h at 100,000×g to separate the microsomal (MF) and supernatant (SF) fractions. For stripping of TH, prior to centrifugation, TH’s were made 1 M with KCl, left to stand for 10 min and centrifuged at 100,000×g for 1 h to separate into MF and SF. MF was re-suspended in the initial volume of PBS plus protease inhibitor cocktail.

### Recombinant Proteins

His-tagged-recombinant Fra-1 (pDS56-HisFra) or c-Fos (pDS56-HisFos) were expressed as described [40], [41] and protein concentration determined by Bradford assay.

### Cell Proliferation Assays

CyQUANT® Cell Proliferation Assay Kit (Invitrogen) was used.

### Electrophoresis and Immunoblot Assays

TH’s (40 µg) fractionated through sodium dodecyl-sulfate-containing 12% polyacrylamide gels were electrotransferred as described [14]. Blocked membranes were incubated overnight at 4°C in PBS-Tween 20 with: rabbit anti-Fra-1 monoclonal antibody (mAb), rabbit anti-c-Fos mAb, mouse anti-PCNA mAb (Santa Cruz Biotechnology, all diluted 1/1000) or mouse anti-α-tubulin DM1A mAb (Sigma-Aldrich, dilution 1/5000). Washed membranes were incubated 2 h at room temperature with secondary goat IRDye 680LT anti-mouse or IRDye 800CW anti-rabbit antibody (1/25000, LI-COR Bioscience, Lincoln, NE, USA), washed and immunodetection performed using ODYSSEY Infrared Imaging System (LI-COR Bioscience).

### Phospholipid Synthesis Determination

*In vitro* phospholipid labeling determinations in tumors, cells and sub-cellular fractions was as described [11] using 100ug of tumor/cell homogenate protein. When stated, recombinant His-tagged Fra-1 or c-Fos (1.5 ng/mg or 1.0 ng/mg of initial TH protein, respectively) were added to assays re-suspended in 300 mM imidazole/8 M urea; control incubates received the same volume of vehicle.

### Immunofluorescence Cell Analysis

Cells grown on acid-washed coverslips coated with poly-Lysine (1g/ml), were treated as described [14]. Briefly, after blocking, coverslips were incubated overnight at 4°C in blocking buffer containing rabbit anti-Fra-1 (dilution 1/500), rabbit anti-c-Fos (1/500), mouse anti-PCNA (1/500) and/or goat anti-calnexin (Santa Cruz Biotechnology, dilution 1/500) antibodies, as indicated. Washed cells (4× in 10 mM PBS, 0.1% Tween 20) were incubated with anti-goat Alexa 546, anti-rabbit Alexa 488 and anti-mouse Alexa 688 (dilution 1/1000, Molecular Probes, Eugene, OR, USA) secondary antibodies for 2 h at RT. Coverslips mounted with FluorSave (Calbiochem, San Diego, CA, USA) were visualized with an Olympus FV1000 or Pascal 5 laser scanning confocal microscope using Olympus (Centre Valley, PA, USA) or Carl Zeiss (St Louis, MO, USA) software for image analysis.

### Immunohistochemistry

Breast Tumor Tissue Array (BioChain Institute Inc., Hayward, CA, USA) specimens were de-waxed and re-hydrated as described [15] and incubated overnight at 4°C with primary antibodies diluted in blocking buffer as follows: rabbit anti-Fra-1, rabbit anti-c-Fos, mouse anti-PCNA and goat anti-calnexin (all 1/300) antibodies. Anti-goat Alexa 546, anti-rabbit Alexa 488 and anti-mouse Alexa 688 secondary antibodies were applied (1/500) and slides mounted with FluorSave (Calbiochem, San Diego, CA, USA). Visualization and image analysis was as described for cells.

### Small Interfering RNA (siRNA) Transfection

To repress human Fra-1 or/and c-Fos, predesigned double-stranded siRNAs (ON-TARGET plus SMART pool; Dharmacon Inc., Lafayette, CO, USA) were used. A siCONTROL non-targeting siRNA pool (Dharmacon Inc.) was used as a negative control. MDA-MB231 cells were grown under standard culture conditions in DMEM supplemented with 10% FBS. After desired cell density and 24 h prior to transfection, cell medium was replaced by free serum and antibiotics medium. Then, cells were transfected with SMART pool siRNA specific for human Fra-1, human c-Fos, both of them, or scrambled siRNA at 25 nM concentrations using Thermo Scientific DharmaFECT 1 transfection reagent (Thermo Fisher Scientific, Lafayette, CO, USA) in Opti-MEM I serum and antibiotics free medium following manufacturer’s instructions. At 96 h incubation, cells were stimulated with complete medium (DMEM supplemented with 10% FBS) and analyzed for proliferation and protein expression.

### Statistical Analysis

Student’s two tailed *t* test of Infostat software (Universidad Nacional de Córdoba, Argentina) was used.

## Results

### Fra-1 and c-Fos Expression is Up-regulated in the Cytoplasm of Growing Beast Cancer Cells and Both Proteins Co-localize with ER Markers

**Figure 1:**
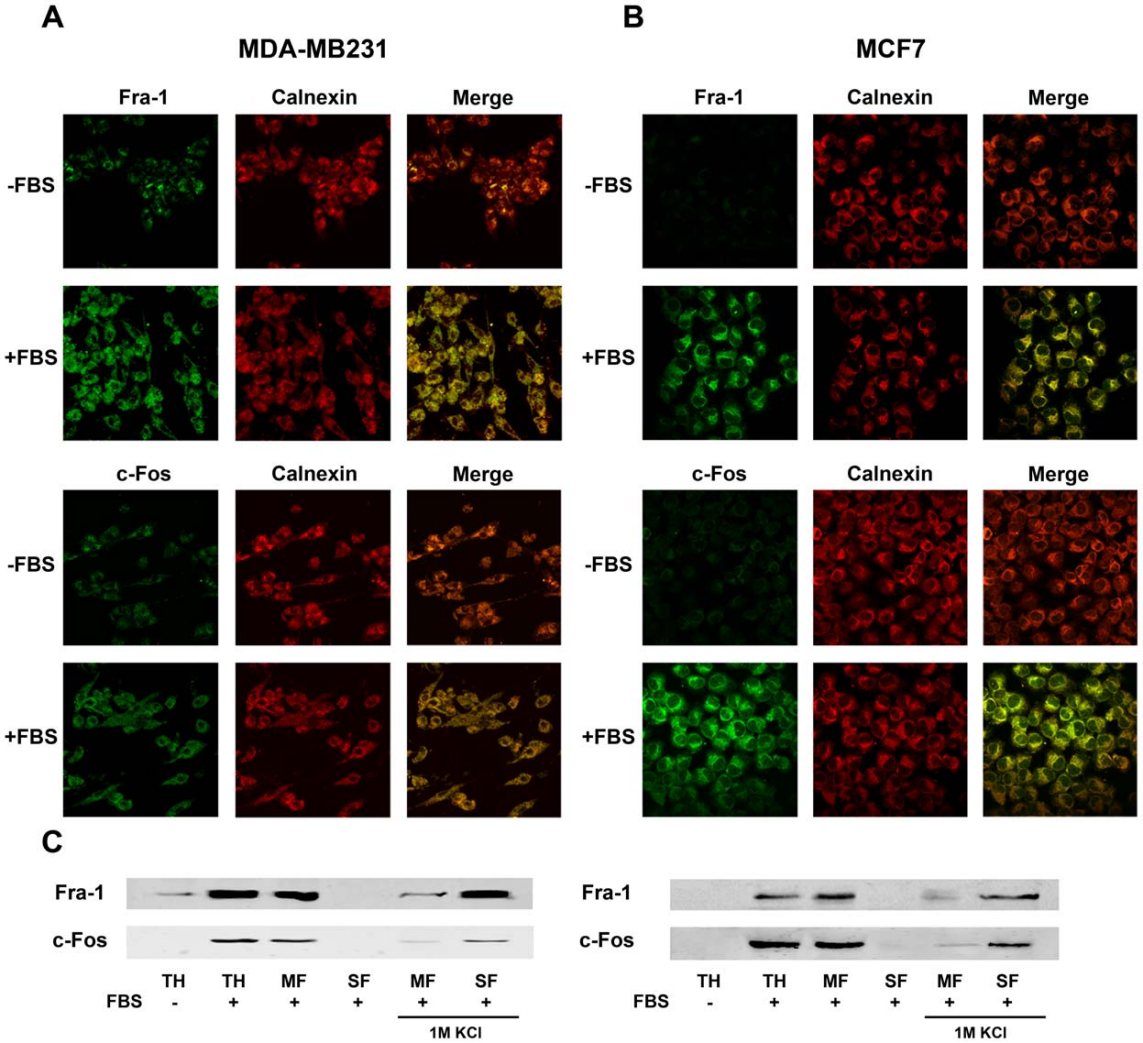
MDA-MB231 and MCF7 cells show abundant Fra-1 and c-Fos expression, which co-localizes with the ER marker calnexin. Expression of Fra-1 (upper left columns), c-Fos (lower left columns) and the ER marker calnexin (middle columns) are shown in MDA-MB231 cells (**A**), and MCF7 cells (**B**). Cells were immunostained for Fra-1 (top panels, green) or c-Fos (lower panels, green) and for calnexin (red). The top rows of each panel show quiescent cells (–FBS) while those in the second row correspond to growing cells (+FBS). The last column of each panel is the merge image of the previous two columns. Yellow color evidences Fra-1/ER (top panels) or c-Fos/ER (lower panels) co-localization sites. Clear co-localization is evidenced for both Fra-1 and c-Fos with the ER marker calnexin as determined by confocal immunofluorescence analysis. (**C**). Fra-1 (upper panel) and c-Fos (lower panel) content was determined by WB in total homogenate (TH) and in the microsomal (MF) and supernatant (SF) fractions obtained after centrifuging MDA-MB231 (left panel) or MCF7 (right panel) cells at 100,000×g for 1 h. In addition, Fra-1 and c-Fos content was examined in MF and SF obtained after stripping MF with 1M KCl prior to centrifugation. Results shown are from one representative experiment out of three performed with essentially the same results.

Fra-1 and c-Fos expression was analyzed by immunofluorescence and immunoblot in MDA-MB231 and MCF7 human breast adenocarcinoma cell lines. MDA-MB231 are highly invasive, Estrogen Receptor α negative, E-cadherin negative and Vimentin positive cells whereas MCF7 are weakly invasive, Estrogen Receptor α positive, E-cadherin positive and Vimentin negative cells [28]. In quiescent MDA-MB231 (Figure 1A) or MCF7 cells (Figure 1B) (–FBS: grown in the absence of FBS), low levels of both Fra-1 (upper panels) and c-Fos (lower panels) were detected. However, upon induction of cells to re-enter growth by feeding with FBS (+FBS), a marked up-regulation of Fra-1 (upper panel) and c-Fos (lower panel) expression was observed in both MDA-MB231 (A) and MCF7 (B) cells.

Interestingly, in growing cells, c-Fos and Fra-1 were present both in the nucleus and in the cytoplasm although both were more abundant in the cytoplasm. When cells were immuno-stained for the ER marker calnexin, the cytoplasmic fraction of both proteins clearly co-localized with the ER marker (Figure 1 A–B, middle columns and merged images to the right). This sub-cellular localization was confirmed by WB after subjecting total homogenate (TH) from quiescent (–FBS) and growing cells (+FBS) to sub-cellular fractionation by centrifugation at 100,000×g for 1 h to isolate the microsomal (MF) and the supernatant (SF) fractions. Both MDA-MB231 (Figure 1C, left panel) and MCF7 cells (Figure 1C, right panel) clearly showed higher Fra-1 and c-Fos immuno-reactivity in total homogenate (TH) from growing cells (+FBS) as compared to quiescent (–FBS) cells and both proteins were recovered in the MF. When the MF was stripped of its associated proteins by treatment with 1M KCl, Fra-1 and c-Fos were no longer recovered in MF but rather were recovered in the SF (Figure 1C, last two lanes).

### Both Fra-1 and c-Fos Activate Phospholipid Synthesis in MDA-MB231 and MCF7 Cells

**Figure 2:**
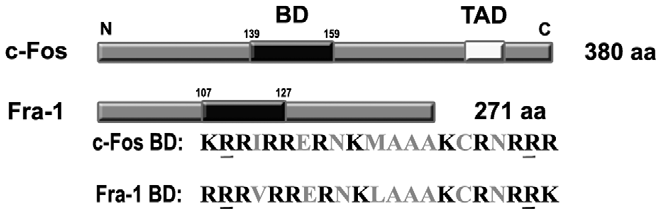
Schematic representations of c-Fos and Fra-1 and comparison of their Basic Domains (BD). c-Fos is a 380 amino acid (aa) protein whereas Fra-1 contains 271 aa. The BD of c-Fos spans from aa 139 to 159 whereas that of Fra-1 spans from aa 107 to aa 127. Comparison of both BDś shows that of the 12 basic aa that each contain (schematized in bold letters), these are highly homologous showing only two conservative substitutions of the basic aa that are underlined in the scheme. c-Fos contains a C-terminal trans-activating domain (TAD) that is not present in Fra-1.

**Figure 3:**
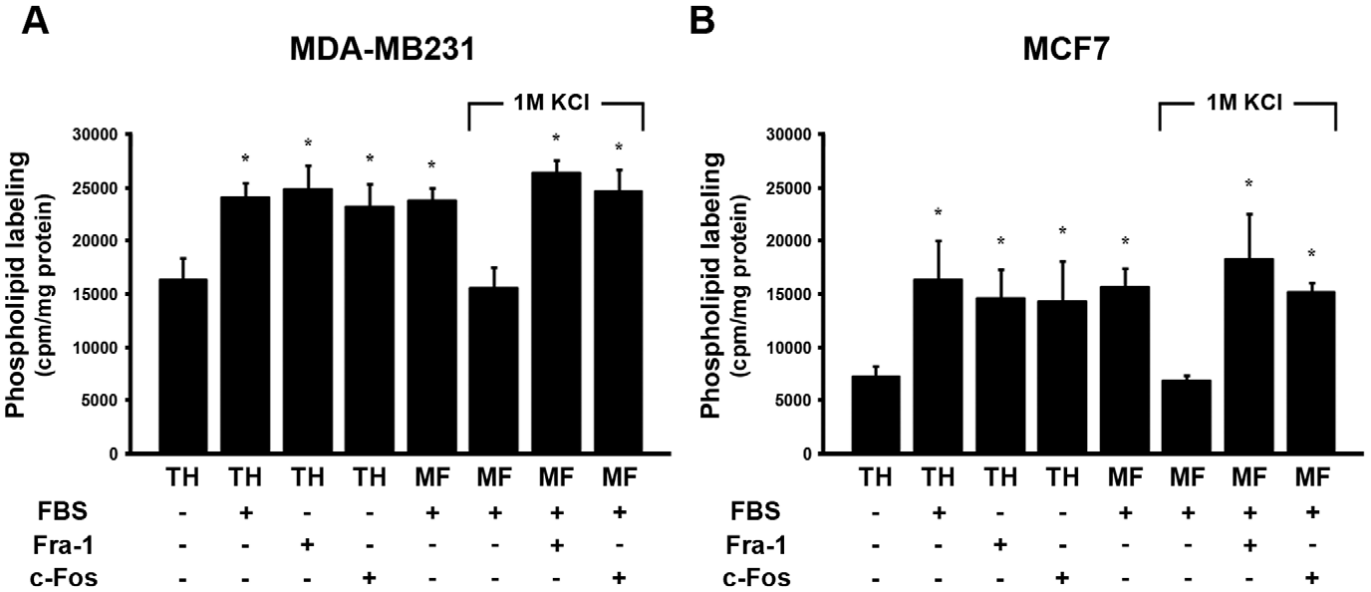
Phospholipid synthesis is activated in membranes from MDA-MB231 and MCF7 cells containing Fra-1 or c-Fos. MDA-MB231 (**A**) and MCF7 (**B**) cells were used to prepare total homogenate (TH), microsomal fraction (MF) and the 1M KCl-stripped microsomal fraction (MF+1M KCl), as indicated under Figure 2. These fractions and the stripped MF plus 1.5 ng of recombinant Fra-1 or 1 ng of c-Fos/µg of TH were assayed for phospholipid synthesis capacity. Results are the mean cpm incorporated into phospholipids/mg protein ± SD of 3 experiments performed in triplicate. *: p<0.01.

We previously showed that the BD of c-Fos (amino acids 139–159) is required to activate phospholipid synthesis [11], [17]. Considering that the BD of Fra-1 and c-Fos are conserved showing only two conservative substitutions in their BD´s (see schematic representation in Figure 2) and that both proteins co-localize with the ER marker calnexin, we next studied if Fra-1, as previously described for c-Fos in other cells [6]–[17], activates phospholipid synthesis in actively growing cells. ^32^P-labeling of phospholipids was determined *in vitro* using TH prepared from quiescent and growing MDA-MB231 and MCF7 cells. TH and the MF of growing MDA-MB231 cells (+FBS, second column) showed a significant increase in ^32^P-phospholipid labeling as compared to quiescent cells (–FBS, first column)(Figure 3A). Furthermore, a comparable activation of ^32^P-phospholipid labeling was observed when recombinant Fra-1 (third column) or c-Fos (fourth column) (1.5 or 1.0 ng/µg of TH protein, respectively) were added to TH of quiescent (–FBS) cells. Similarly, when the TH from growing cells (+FBS) was stripped with 1M KCl (thus containing negligible levels of c-Fos and Fra-1 as shown in Figure 1), ^32^P-phospholipid labeling decreased markedly mirroring the levels in TH from quiescent cells. Addition of recombinant Fra-1 or c-Fos to these stripped membranes restored ^32^P-phospholipid synthesis to the initial activated levels (last two columns). Similar results were observed with MCF7 cells (Figure 3B). Altogether, these results show that Fra-1 is capable of activating phospholipid synthesis to levels comparable to those obtained with c-Fos.

### Blocking Fra-1 and c-Fos Abrogates Phospholipid Synthesis Activation and Cell Proliferation

**Figure 4:**
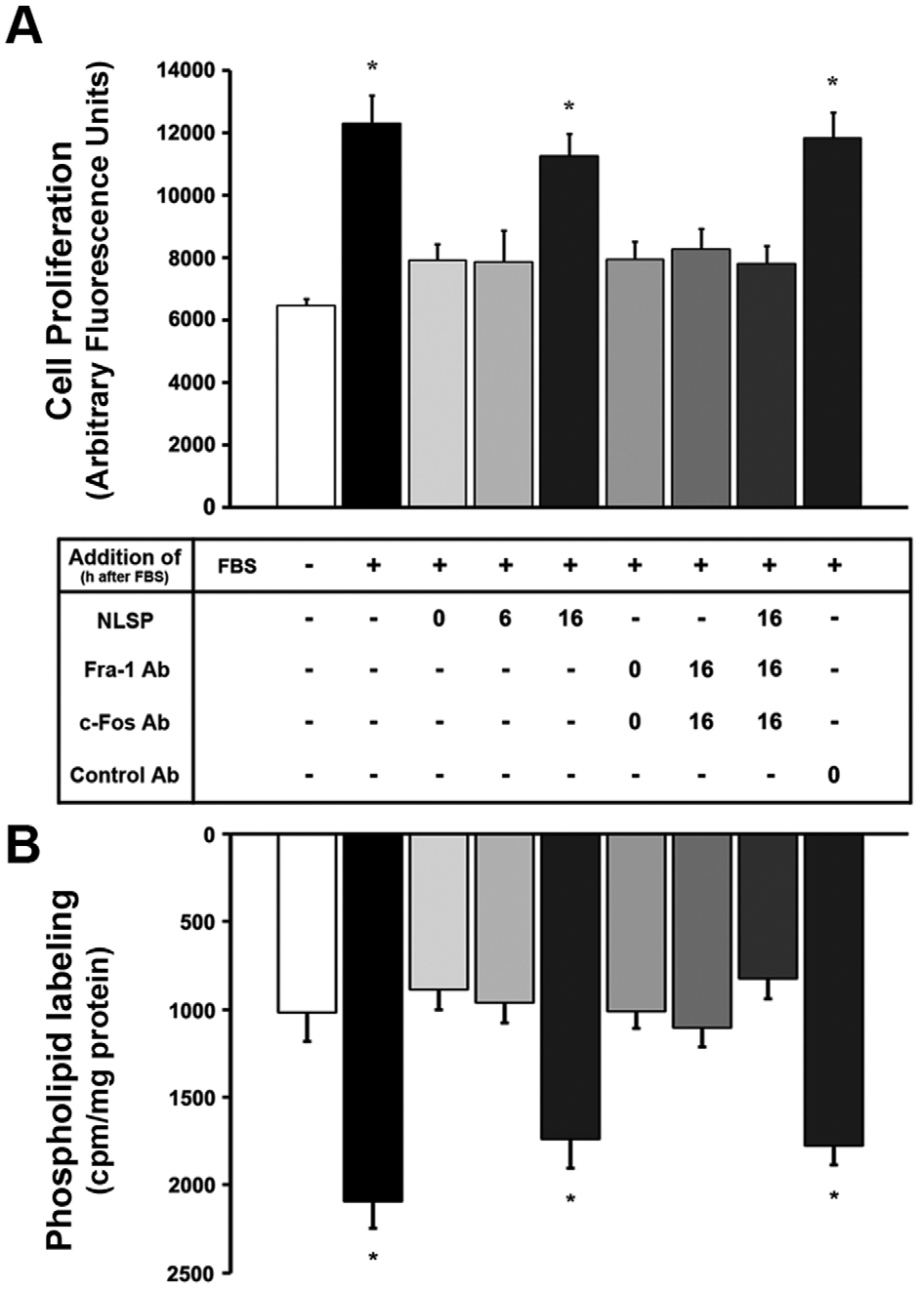
Nuclear AP-1-Fra-1/c-Fos and cytoplasmic Fra-1/c-Fos are required at early and late stages of cell proliferation, respectively. (**A**) MDA-MB231 cells were cultured+or – NLSP that blocks AP-1 nuclear import, in the presence or the absence of anti-Fra-1 or anti-c-Fos antibodies added at the indicated times (0 h or 16 h) after FBS addition and examined for proliferation. Controls were performed that received FITC-IgG antibody (last column). Proliferation was determined as indicated under Materials and Methods and expressed as arbitrary fluorescent units of DNA. Results are the mean ± SD of 3 experiments performed in quintuplicate; 10,000 cells were plated for each proliferation assay. Note that nuclear import of Fra-1/c-Fos-AP-1 is required only during the first 16 h after FBS addition whereas cytoplasmic Fra-1/c-Fos are required at all time points (compare column 5 with columns 6,7 or 8). (**B**) TH form MDA-MB231 cells cultured as in (A) were assayed for phospholipid synthesis capacity. Results are the mean cpm incorporated into phospholipids/mg protein ± SD of 3 experiments performed in triplicate. *: p<0.001.

The participation of nuclear and cytoplasmic c-Fos in driving tumor cell proliferation and growth was examined in growing MDA-MB231 cells cultured in the presence or the absence of an AP-1 Nuclear Localization Sequence Peptide (NLSP) that blocks the nuclear import of c-Fos and Fra-1 as AP-1 dimers [42]. Figure 4A shows that after culturing cells +FBS for 36 h, cell number roughly doubled. However, as described previously [14], adding NLSP to the cultures at 0 or 6 h after priming cells to proliferate (+FBS) blocks cell proliferation, whereas at later times (16 h) it is no longer effective (column 5), indicating that nuclear AP-1-c-Fos/Fra-1 is required to trigger proliferation at early stages. By contrast, cytoplasmic c-Fos and Fra-1 are required at all time points because blocking their activity with specific neutralizing antibodies delivered at any time after feeding FBS,+or – NLSP, specifically blocks proliferation (Figure 4A, columns 6,7,8). These results highlight the need of AP-1 to trigger the genomic events for proliferation and growth and that of cytoplasmic Fra-1 and c-Fos to activate lipid synthesis required for membrane biogenesis to sustain growth.

To confirm the importance of Fra-1/c-Fos-dependent phospholipid synthesis activation during proliferation and growth, this was assessed in FBS-stimulated MDA-MB231 cells cultured +NLSP or -NLSP and transfected with c-Fos and Fra-1 neutralizing antibodies at different times. Accordingly, higher rates of ^32^P-phospholipid labeling were detected in growing cells (+FBS) when compared to quiescent cells (–FBS) (Figure 4B). Feeding cells with NLSP at 0 h or 6 h after FBS priming, abrogated ^32^P-phospholipid labeling activation, whereas primed cells-activated levels were observed when NLSP was added at 16 h. These results further support that nuclear AP-1 is needed to trigger the genomic events for proliferation and growth whereas cytoplasmic Fra-1 and c-Fos are required to sustain growth.

**Figure 5:**
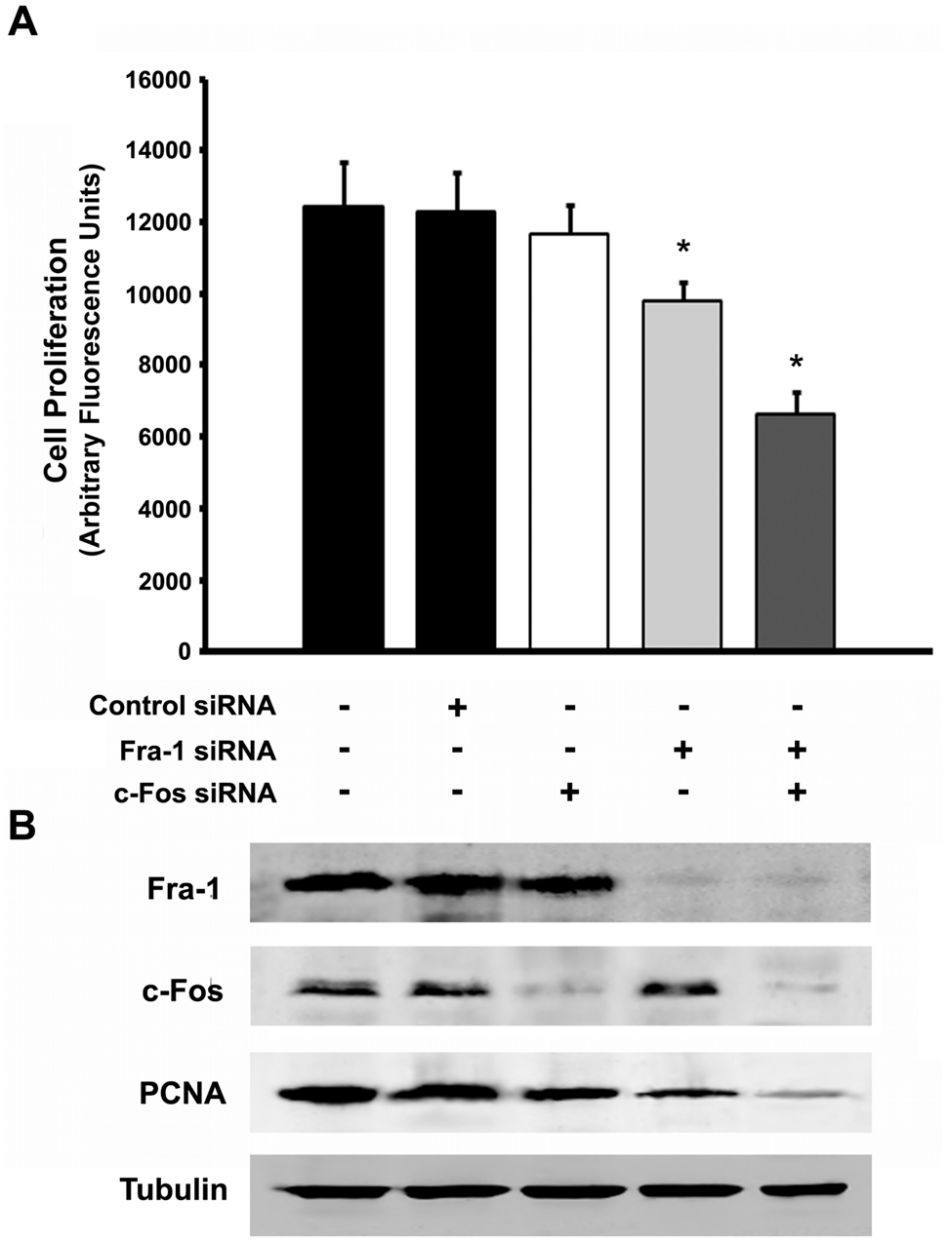
Both Fra-1 and c-Fos are capable of sustaining cell proliferation. (**A**). MDA-MB231 cells non-transfected (first column), control-transfected (second column) or transfected to block the expression of c-Fos (third column), or of Fra-1 (fourth column), or of both proteins (last column) were examined for cell proliferation. Proliferation was determined as indicated under Materials and Methods and expressed as arbitrary fluorescent units of DNA. Results are the mean ± SD of 3 experiments performed in sextuplicate; 10,000 cells were plated for each proliferation assay. *: p<0.001. (**B**) WB determination of the expression of Fra-1 (top row), c-Fos (second row), PCNA (third row) and Tubulin used as a loading control (bottom row) of the samples used in (A) to determine cell proliferation.

Taking into consideration that MDA-MB231 and MCF7 cells contain both c-Fos and Fra-1, it was deemed of interest to determine if both proteins are required to support cell proliferation or if one is enough and can substitute for the other. For this, quiescent MDA-MB231 cells were transfected with siRNA against c-Fos or against Fra-1 or both. At 96 h of transfection, cells were induced to proliferate by the addition of FBS to the culture medium and proliferation determined 36 h later. Figure 5A shows that proliferation of mock-transfected cells was essentially the same as that of non-transfected cells whereas transfection of siRNA against either c-Fos or Fra-1 partially blocked proliferation. However, blocking the expression of both proteins completely blocks proliferation indicating that either c-Fos or Fra-1 can support proliferation provided that protein expression levels are high enough. Fig. 5B shows that treatment of cells with siRNA effectively blocked c-Fos and Fra-1 expression as determined by WB assays.

### Malignant Human Breast Tumors Show Abundant Fra-1 and c-Fos Expression Associated to the ER and Activated Phospholipid Synthesis

**Figure 6:**
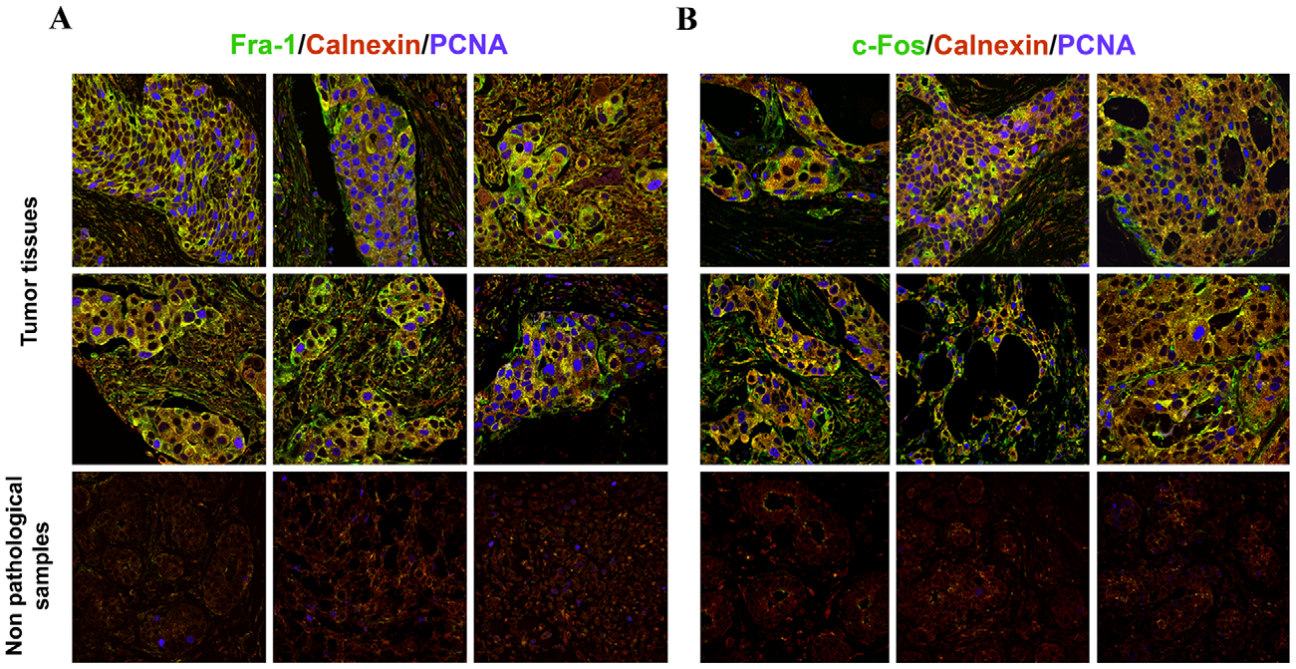
Human malignant breast carcinomas show abundant Fra-1 and c-Fos expression co-localizing with the ER marker calnexin. Expression of Fra-1 (**A**), c-Fos (**B**), the ER marker calnexin, and the nuclear marker of proliferating cells PCNA was examined by triple labeling in malignant human breast tumor specimens (n = 210) and non-pathological samples (n = 37). Six representative samples of invasive ductal carcinomas from a tissue array immunostained for Fra-1 (A, green) or c-Fos (B, green), calnexin (red) and PCNA (blue) and three non-pathological samples (bottom row) are shown for each onco-protein examined. Note that all actively proliferating cells (PCNA positive cells) show abundant Fra-1 and c-Fos expression which in all cases is observed co-localizing with the ER marker as evidenced by the yellow color of the merged, triple-labeled images.

Considering the results described above, we analyzed Fra-1 and c-Fos expression and phospholipid synthesis activation capacity in malignant human breast tumor samples. Immunohistochemistry assays revealed a marked over-expression of Fra-1 and c-Fos in 100% of 210 tissue samples from different human breast tumors [invasive ductal carcinoma (n = 200), Medullary carcinoma (n = 2), Phyliodes sarcoma (n = 2), Mucinous carcinoma (n = 2), lobular carcinoma in situ (n = 2) and squamous cell carcinoma (n = 2)], contrasting with the low or undetectable levels of Fra-1 and c-Fos detected in the non-pathological samples (n = 37). Noteworthy, both proteins were over-expressed in 95% of the tumor samples analyzed, which also showed significant PCNA staining which evidenced their active proliferative status. When assessing the subcellular localization of these proteins, Fra-1 was found mainly in the cytoplasm: 69% of tumor samples showed only cytoplasmic Fra-1 whereas the remaining tumor samples contained both nuclear and cytoplasmic-localized Fra-1 (31%). c-Fos was also preferentially present in the cytoplasm of the tumor samples: 100% showed cytoplasmic c-Fos whereas, of these, 63% also contained nuclear c-Fos. Remarkably, in all cases cytoplasmic Fra-1 and c-Fos co-localized with the ER marker calnexin (Figure 6A, B).

**Figure 7:**
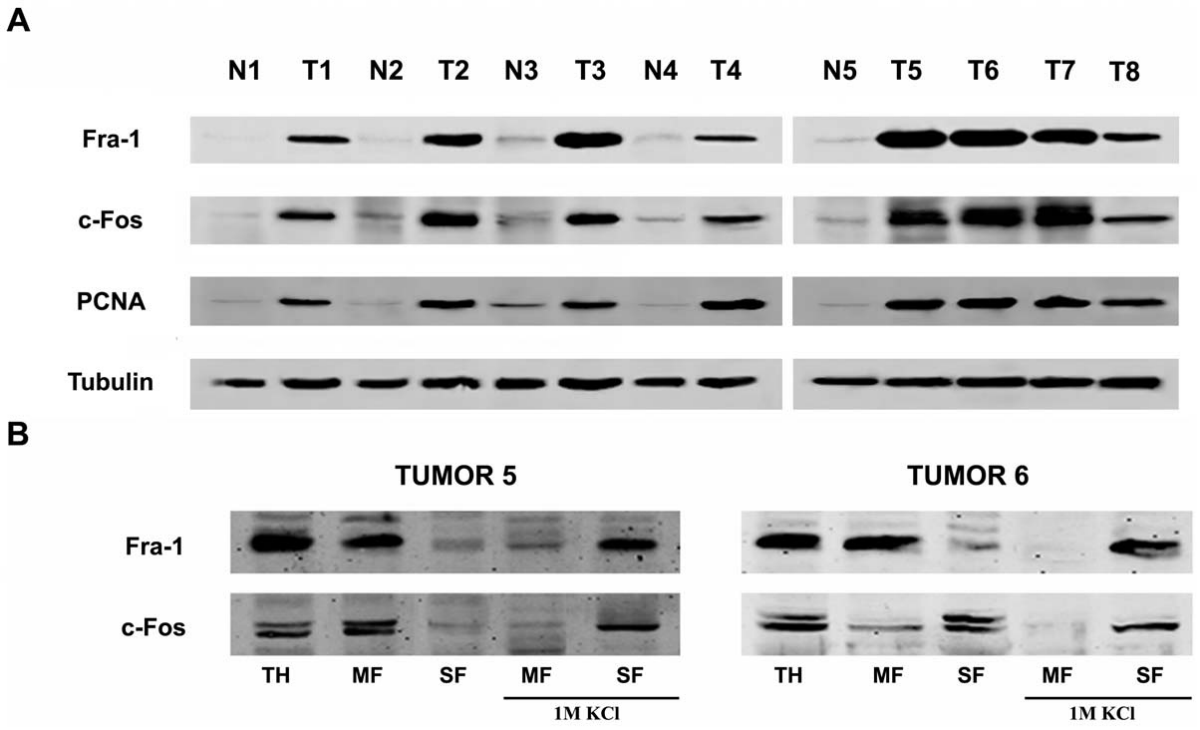
Fra-1 and c-Fos expression in malignant breast tumor biopsies and non-pathological samples. (**A**). Fra-1 (first row), c-Fos (second row) and PCNA content was determined by WB in total homogenate prepared from 5 normal (N1 to N5) and 8 tumor samples (T1 to T8) randomly selected from the collection of breast tissue samples. Note the abundant Fra-1, c-Fos and PCNA expression in all tumor samples contrasting with the very low or undetectable expression levels in the non-pathological ones. Tubulin was used as a loading control (lower panel). (**B**) TH, MF and SF obtained as described under Figure 2 from tumors #5 (left panel) and #6 (right panel) were examined for Fra-1 and c-Fos content. In addition, Fra-1 and c-Fos content was examined in MF and SF obtained after stripping MF with 1M KCl prior to centrifugation. Results shown are from one representative experiment out of three performed with essentially the same results.

Figure 7A shows immunoblot experiments of 5 normal (N1 to N5) and 8 tumor samples (T1 to T8) randomly selected from the breast tissue collection. Fra-1 and c-Fos over-expression is clearly observed in actively proliferating tumor samples (as evidenced by the high levels of PCNA immuno-reactivity) as compared to their non-pathological counterparts. Tubulin staining was used as a loading control (Figure 7A). Subjecting tumor samples 5 and 6 to subcellular fractionation and WB confirmed that Fra-1 and c-Fos expression is associated to MF and that this association is reversed by 1M KCl-treatment (Figure 7B).

### Fra-1 and c-Fos Activate Phospholipid Biosynthesis in Human Breast Tumors

**Figure 8:**
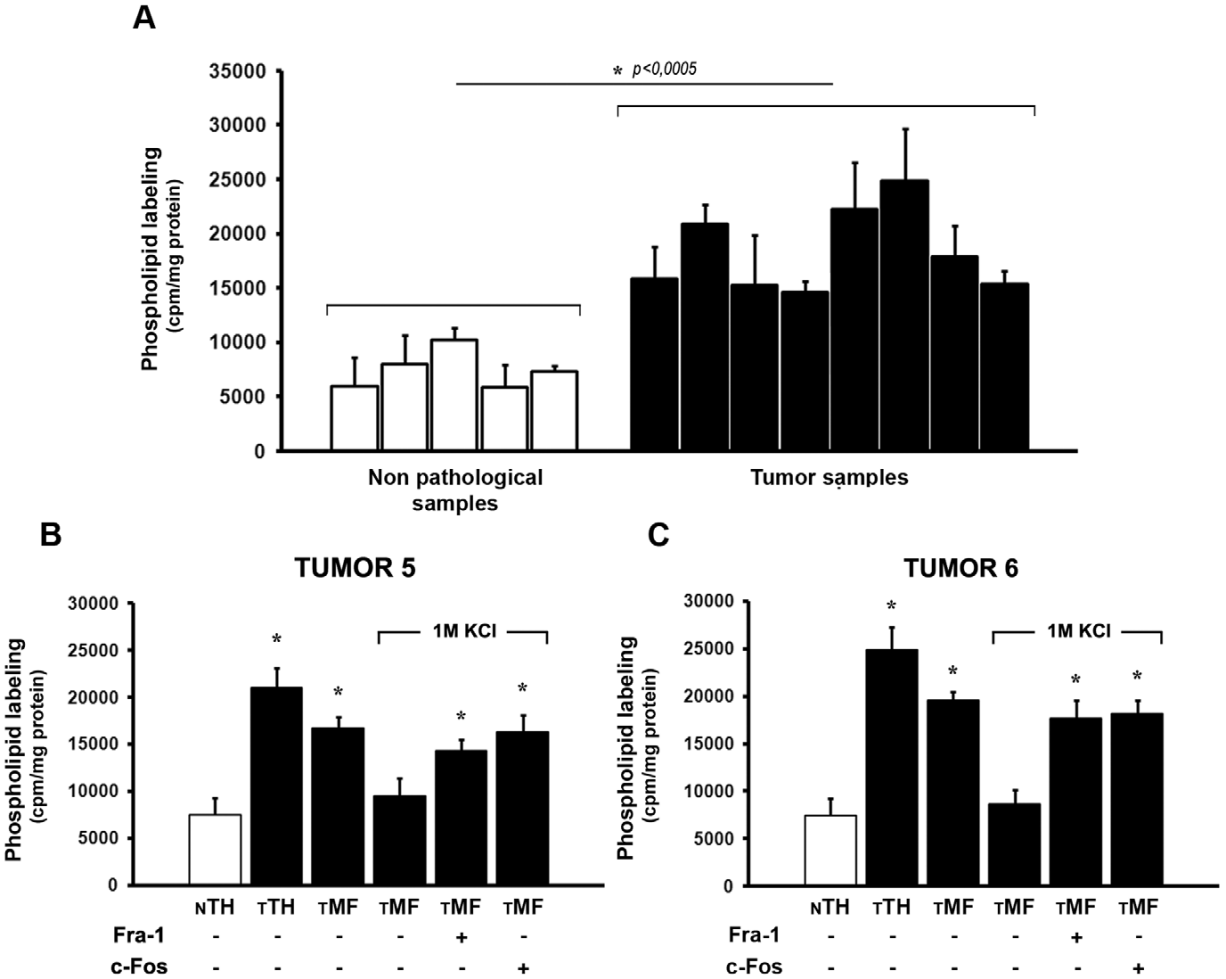
Phospholipid synthesis is activated in Fra-1 or c-Fos-containing human breast tumor biopsies as compared to non-pathological specimens. (**A**) Phospholipid labeling capacity was determined in TH prepared from the 5 non-pathological (white columns) and 8 tumor specimens (black columns) shown in Figure 7. Results are the mean cpm incorporated into phospholipids/mg protein ± SD of 2 experiments performed in triplicate with each sample. (**B** and **C**) Black columns show the phospholipid labeling capacity (mean cpm incorporated into phospholipids/mg TH protein ± SD of 2 experiments performed in triplicate) of tumor samples #5 (**B**) and #6 (**C**) in TH (TTH), MF (TMF), 1M KCl-stripped MF (TMF+1M KCl) and stripped MF plus 1.5 ng of recombinant Fra-1 or 1 ng of c-Fos/µg of TH. White columns (NTH) show the phospholipid labeling capacity of pooled TH from the 5 non-pathological samples shown in (A). *: p<0.01.

^32^P-Phospholipid labeling was assayed *in vitro* using fresh human breast tumor and non-pathological samples. Figure 8A shows high ^32^P-phospholipid labeling in TH from all the tumor samples as compared to the mean value from the non-pathological samples (Figure 8A). This elevated phospholipid synthesis correlates with the elevated content of c-Fos/Fra-1 and the highly proliferative status of tumor cells (Figure 7A). Subcellular fractionation of tumors 5 and 6 confirmed that the activated-^32^P-phospholipid labeling capacity was preponderantly present in MF and that this activity was abrogated when membranes were stripped with 1M KCl (Figure 7B–C, fourth columns). Both, Fra-1 and c-Fos activate phospholipid synthesis as the addition of either Fra-1 or c-Fos to these stripped membranes restores phospholipid synthesis to the initial rates observed in the untreated tumor MF (Figure 8B–C). These results confirm data obtained with MDA-MB231 and MCF7 cells and further support the role of Fra-1 and c-Fos as activators of phospholipid synthesis to support membrane biogenesis for tumor cell growth.

## Discussion

Fra-1 is a Fos family member that is over-expressed in diverse types of human cancers including brain [37], [43], lung [44], oesophagus [36], thyroid [38], colorectal, skin, ovary, etc. (reviewed in [24], [27]). Of the four Fos family members, Fra-1 is likely to be the most frequently expressed in different forms of human cancer [24], [27]. Several studies have shown that Fra-1 over-expression in breast tumor cell lines is associated with an aggressive behavior of these cells [28], [45]. Although only a few reports have addressed Fra-1 over-expression in clinical tumors, this was found in all malignant breast tissues [31], [33], [34]. Furthermore, in all these studies, nuclear and cytoplasmic Fra-1 was found although in many cases only nuclear-containing Fra-1 cells were considered as Fra-1-positive cells [34]. It was reasoned that only nuclear-localized Fra-1 is important due to its transcription factor function, disregarding the possibility of other functions for this protein. By contrast, Song *et al.* [31] found that 90.2% of all breast carcinomas analyzed (n = 445) showed nuclear and cytoplasmic Fra-1 over-expression whereas only 9.2% of the samples (n = 41) showed only nuclear Fra-1 over-expression; of the 20 benign tumors examined, only 3 showed weak cytoplasmic immuno-reactivity that co-existed with the nuclear reactivity that was present in all samples. However, a clear shift from nuclear to a simultaneous nuclear/cytoplasmic localization of Fra-1 was noticed in ∼90% of breast carcinomas examined which led them to conclude that a non-transcriptional function of Fra-1 remained to be demonstrated [31].

We previously showed that over-expressed, cytoplasmic c-Fos activates phospholipid synthesis in brain tumor cell lines and in human brain tumors thus supporting the elevated rates of membrane biogenesis required for the exacerbated growth of these tumors [15]. To activate phospholipid synthesis, the basic domain of c-Fos (aa 139–160) is required [16], [17]. Furthermore, point mutations of this conserved BD in basic amino acids #139 or #144 (K139N, R144N) has no effect on BD’s lipid synthesis activating capacity whereas that of basic amino acid #146 (R146N) completely abolishes this activity further supporting the importance of BD for the activation of specific enzymes in the pathway of synthesis of lipids [17]. Consequently, considering the results described above regarding Fra-1 cytoplasmic over-expression and the fact that Fra-1 and c-Fos share their basic domains, we herein addressed the possibility that Fra-1 may also activate phospholipid synthesis in growing human breast tumors. Using breast tumor cell lines and clinical tumor samples, it is demonstrated that this is indeed the case. Briefly, we found that growing MDA-MB231 and MCF7 cells exhibit high rates of phospholipid biosynthesis as compared with quiescent cells. Furthermore, stripping membranes prepared from growing cells of its non-integral proteins by 1M KCl-treatment resulted in quiescent cell phospholipid synthesis rates, which was restored to its initial activated rates by the addition of recombinant Fra-1 or c-Fos to the assay medium. These results were mirrored when analyzing human breast tumor samples: phospholipid synthesis was also significantly higher in tumors as compared to normal tissue and was significantly reduced when subjecting tumor samples to 1M KCl-treatment. Addition of Fra-1 or c-Fos to stripped samples restored phospholipid synthesis activation to initial rates. Finally, the previously shown dual function (nuclear and cytoplasmic) of c-Fos (14,11) was also demonstrated for Fra-1 in experiments in which cells were fed NLSP at different times after inducing them to re-enter growth. AP-1-Fra-1 and/or AP-1-c-Fos were required to trigger proliferation only at early stages of cell proliferation. In contrast, at later stages of cell proliferation (16 h after priming cells), cytoplasmic Fra-1 and/or c-Fos (that activate phospholipid biosynthesis) were necessary to sustain proliferation. Blocking Fra-1 and c-Fos with intracellular neutralizing antibodies abrogated both phospholipid synthesis activation and cell proliferation.

The cytoplasmic regulatory role of c-Fos or Fra-1 is not the only AP-1 independent activity of an IEG. c-Jun was also shown to protect cells from apoptosis independently of its AP-1 activity [46]. In addition, cytoplasmic p53 is capable of triggering apoptosis in the absence of transcription [47] whereas translocation of nuclear p21Cip1/WAF1 to the cytoplasm promotes resistance to apoptotic stimuli [48]. Consequently in light of these results and of ours shown herein, a new concept is emerging in which the biological activity of these proteins results from the combination of their nuclear and cytoplasmic activities [49]. These data may highlight the significance of therapy based on the blockade of Fra-1 and/or c-Fos functions in breast cancer. Therapies directed towards blocking Fra-1 and c-Fos expression are promising moreover if it is considered that these proteins are normally down-regulated in normal breast cell growth but become up-regulated during, and are causally related to, breast cancer progression.
